# Systemic Inflammatory Biomarkers Define Specific Clusters in Patients with Bronchiectasis: A Large-Cohort Study

**DOI:** 10.3390/biomedicines10020225

**Published:** 2022-01-21

**Authors:** Xuejie Wang, Carmen Villa, Yadira Dobarganes, Casilda Olveira, Rosa Girón, Marta García-Clemente, Luis Máiz, Oriol Sibila, Rafael Golpe, Rosario Menéndez, Juan Rodríguez-López, Concepción Prados, Miguel Angel Martinez-García, Juan Luis Rodriguez, David de la Rosa, Xavier Duran, Jordi Garcia-Ojalvo, Esther Barreiro

**Affiliations:** 1 Lung Cancer and Muscle Research Group, Pulmonology Department, Hospital del Mar-IMIM, Parc de Salut Mar, PRBB, C/Dr. Aiguader, 88, 08003 Barcelona, Spain; Xuejie.Wang@e-campus.uab.cat; 2 Department of Medicine, Universitat Autònoma de Barcelona (UAB), 08035 Barcelona, Spain; 3 Respiratory Department, Clínica Fuensanta, 28015 Madrid, Spain; carmenvilla@neumo.es (C.V.); Dogar_yad@clinicmadrid.org (Y.D.); 4 Respiratory Department, Hospital Regional Universitario de Málaga, 29003 Málaga, Spain; casi1547@separ.es; 5 Instituto de Investigación Biomédica de Málaga (IBIMA), Universidad de Málaga, 29003 Málaga, Spain; 6 Respiratory Department, Instituto de Investigación Sanitaria, Hospital Universitario de la Princesa, 28015 Madrid, Spain; rosamaria.giron@salud.madrid.org; 7 Respiratory Department, Hospital Universitario Central de Asturias, 33071 Oviedo, Spain; marta.garciac@sespa.es; 8 Respiratory Department, Hospital Ramon y Cajal, 28015 Madrid, Spain; luis.maiz@salud.madrid.org; 9 Respiratory Department, Hospital Clínic, 08035 Barcelona, Spain; OSIBILA@clinic.cat; 10 Centro de Investigación en Red de Enfermedades Respiratorias (CIBERES), Instituto de Salud Carlos III (ISCIII), 28015 Madrid, Spain; martinez_miggar@gva.es; 11 Respiratory Department, Hospital Lucus Augusti, 27080 Lugo, Spain; rafa898@separ.es; 12 Respiratory Department, Hospital Universitario y Politécnico La Fe, 46003 Valencia, Spain; Menendez_ros@gva.es; 13 Respiratory Department, Hospital San Agustin, 33401 Avilés, Spain; juan.rodriguez@sespa.es; 14 Respiratory Department, Hospital la Paz, 28015 Madrid, Spain; mconcepcion.prados@salud.Madrid.org; 15 Respiratory Department, Hospital Clínico San Carlos, 28015 Madrid, Spain; jlrodr01@ucm.es; 16 Instituto de Investigación Sanitaria del Hospital Clínico San Carlos (IdISSC), 28015 Madrid, Spain; 17 Departamento de Medicina, Universidad Complutense de Madrid, 28015 Madrid, Spain; 18 Respiratory Department, Hospital Santa Creu I Sant Pau, 08035 Barcelona, Spain; drosa@santpau.cat; 19 Scientific and Technical Department, Hospital del Mar-IMIM, 08035 Barcelona, Spain; xduran@imim.es; 20 Department of Health and Experimental Sciences (CEXS), Universitat Pompeu Fabra (UPF), 08035 Barcelona, Spain; jordi.g.ojalvo@upf.edu

**Keywords:** non-cystic fibrosis bronchiectasis, blood neutrophil, eosinophil, lymphocyte counts, C reactive protein, hemoglobin, hierarchical clustering, phenotypic clusters, multivariate analyses, clinical outcomes, disease severity scores

## Abstract

Differential phenotypic characteristics using data mining approaches were defined in a large cohort of patients from the Spanish Online Bronchiectasis Registry (RIBRON). Three differential phenotypic clusters (hierarchical clustering, scikit-learn library for Python, and agglomerative methods) according to systemic biomarkers: neutrophil, eosinophil, and lymphocyte counts, C reactive protein, and hemoglobin were obtained in a patient large-cohort (*n* = 1092). Clusters #1–3 were named as mild, moderate, and severe on the basis of disease severity scores. Patients in cluster #3 were significantly more severe (FEV_1_, age, colonization, extension, dyspnea (FACED), exacerbation (EFACED), and bronchiectasis severity index (BSI) scores) than patients in clusters #1 and #2. Exacerbation and hospitalization numbers, Charlson index, and blood inflammatory markers were significantly greater in cluster #3 than in clusters #1 and #2. Chronic colonization by *Pseudomonas aeruginosa* and COPD prevalence were higher in cluster # 3 than in cluster #1. Airflow limitation and diffusion capacity were reduced in cluster #3 compared to clusters #1 and #2. Multivariate ordinal logistic regression analysis further confirmed these results. Similar results were obtained after excluding COPD patients. Clustering analysis offers a powerful tool to better characterize patients with bronchiectasis. These results have clinical implications in the management of the complexity and heterogeneity of bronchiectasis patients.

## 1. Introduction

Bronchiectasis is a chronic respiratory disease characterized by a permanent dilatation of the airways of different etiology [1,2,3]. Bronchiectasis is a very heterogeneous and complex disease, in which patients experience recurrent exacerbations mainly due to bronchial infection. Exacerbations negatively impact on the patients’ quality of life and disease prognosis [4].

The immune response against infections is crucial in bronchiectasis patients [5]. Neutrophilic inflammation is the predominant phenotype in these patients. In response to bacterial loads, neutrophils are recruited to the lungs, where they secrete antimicrobial peptides to fight against infection [6]. However, other inflammatory cell types such as eosinophils are also involved in the pathobiology of bronchiectasis, particularly in the response to different biological agents [7,8,9,10] as well as to inhaled corticosteroids [11,12]. Lymphocytic infiltration was also demonstrated to take place just beneath the basement membrane of the epithelium in bronchiectasis patients [13].

Nutritional abnormalities and systemic inflammation are common in patients with bronchiectasis, particularly in male patients compared to females, as demonstrated in a recent study [14]. Moreover, body composition and systemic inflammation have a prognosis value in other chronic respiratory conditions such as chronic obstructive pulmonary disease (COPD) [15,16,17]. Disease severity scores usually take into account variables that reflect the status of the lung disease, but not those involving the systemic components. This imposes a challenge in the overall assessment and phenotyping of the patients.

Thus, phenotypic clustering in which variables related to systemic manifestations and inflammatory parameters are analyzed jointly may be of interest in clinical decision processes of patients with bronchiectasis. In this regard, results obtained from clinical tests can be analyzed using different approaches, in which software tools are applied to complex clinical and biological data sets obtained from large cohorts of patients. Phenotypic classification of bronchiectasis patients according to several clinical and biological markers may help predict disease severity, the risk of exacerbations, and disease prognosis. Recent investigations showed that a cut-off value greater than 5 reliably predicted hospitalizations and all-cause mortality according to the FACED (FEV_1_, age, chronic colonization, radiological extension, and dyspnea) and bronchiectasis severity index (BSI) scores [18] and that eosinophil levels defined differential clinical clusters of bronchiectasis patients [19].

The current investigation sought to tease out differential phenotypic characteristics of patients with bronchiectasis using data mining approaches on the basis of the following systemic inflammatory and nutritional parameters: blood neutrophil, eosinophil, and lymphocyte counts, C reactive protein, and hemoglobin in a large-cohort of patients from the Spanish Online Bronchiectasis Registry (RIBRON) [19,20]. Three clusters of patients with differential clinical phenotypes were obtained. Thus, the study objectives were: (1) to identify different clusters of patients included in this registry that could discriminate differential phenotypes on the basis of blood neutrophil, eosinophil, and lymphocyte counts along with C reactive protein and hemoglobin levels, (2) to analyze potential differences between the clusters in several clinical parameters involving the assessment of lung function, nutritional status, and a general clinical evaluation, and (3) to stratify the clusters according to disease severity following the exacerbation FACED (EFACED), FACED, and BSI indices.

## 2. Methods

### 2.1. Study Design

This was a multicenter, prospective, and observational study, in which 43 centers from Spain participated within the frame of the RIBRON database between February 2015 and October 2019 [21,22,23]. Strengthening the Reporting of Observational studies in Epidemiology (STROBE) reporting guidelines were used to design the current investigation [24]. The quality of the data introduced in the registry was always monitored and ensured by an external contract research organization (CRO).

### 2.2. Study Population

**Figure 1:**
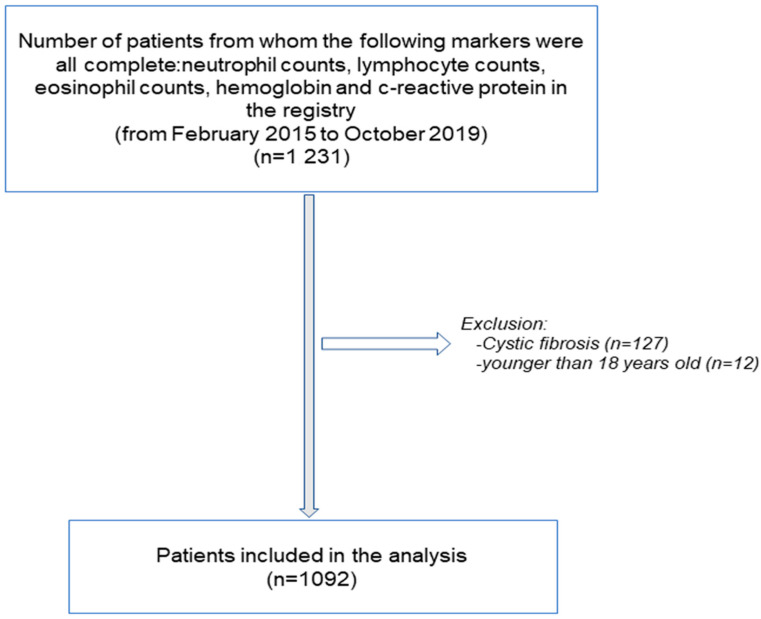
Flow-chart of the study.

The patient recruitment flow-chart is depicted in Figure 1. Inclusion criteria were as follows: adult patients who had been diagnosed with non-CF bronchiectasis as a result of a high-resolution computerized tomography (HRCT) [21,23,25,26,27,28]. The participants were stable patients and did not report any acute exacerbation at least in the last four weeks prior to study entry. A total of 1092 patients were analyzed from the registry. Study variables were as follows: anthropometry, smoking history, lung function, hemogram, inflammatory blood cells, and nutritional parameters were analyzed using custom data-analysis software tools. The exclusion criteria were the following: traction bronchiectasis and/or cystic fibrosis (sweat chloride test and/or genetic confirmation), and age younger than 18 years old.

The World Medical Association for Research in Humans (Seventh revision of the Declaration of Helsinki, Fortaleza, Brazil, 2013) guidelines were followed in the study [29]. Ethics approval was obtained from the Ethics Committee at the Hospital Josep Trueta Girona (# 001-2012, Hospital Universitari Dr. Josep Trueta, Girona, Spain) from all the participants. All the patients signed the informed written consent to participate in the registry. The information remained confidential at all times and no personal information related to any of the participants was introduced in the registry.

### 2.3. Study Variables and Scores

Etiology of the non-CF bronchiectasis, anthropometry (age, sex, and body mass index), lung function, exercise capacity, chronic colonization by *Pseudomonas aeruginosa*, chronic colonization with other microorganisms, radiologic extension, dyspnea, the number of exacerbations and hospitalizations for exacerbations in the previous year, the Charlson index, smoking history, nutritional status, and systemic inflammatory cells and markers were obtained from all the patients. FACED [30], EFACED [31], and bronchiectasis severity index (BSI) [32] were calculated on the basis of the clinical study variables.

### 2.4. Patient Clustering

The study population was clustered into three major groups of patients on the basis of the following analytical parameters: eosinophils, neutrophils, lymphocytes, C-reactive protein (CRP), and hemoglobin that correlated with EFACED score. A total of 1092 patients met the criteria for these analyses (all these patients had valid values for all five parameters). A hierarchical clustering was performed on the basis of the five biomarkers in the 1092 patients using the scikit-learn library for Python [33]. Agglomerative methods, in which clusters start by a single patient and are subsequently merged and fused from the previous steps into bigger clusters, were used in this study. The criterion of such fusion is the minimal distance between the two clusters to be fused, as specified by the chosen linkage function. The algorithm stopped when the number of clusters fell to 5, which is the level that produced an optimal classification of the patients according to the EFACED score (Table 1). As seen in Table 1, cluster # 3 was very small and was indistinguishable from cluster # 2 (EFACED score 3.57 in both clusters). In a similar fashion, clusters # 1 and # 4 were similar as no statistically significant difference was observed in EFACED score (2.60 versus 2.88, respectively). Hence, the 1092 patients were finally subdivided into three major clusters 1–3 that were labeled as mild (*n* = 242 patients), moderate (*n* = 515 patients), and severe (*n* = 335 patients) clusters of patients (Table 1).

**Figure 2:**
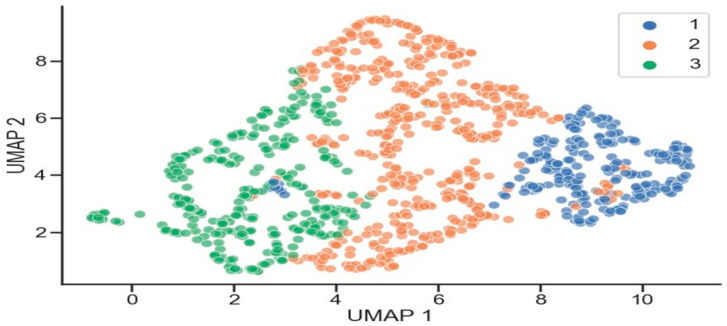
Uniform Manifold Approximation and Projection (UMAP) representation of the three clusters of patients. Three clusters of patients: blue, light brown, and green represent mild, moderate, and severe bronchiectasis patients, respectively.

**Figure 3:**
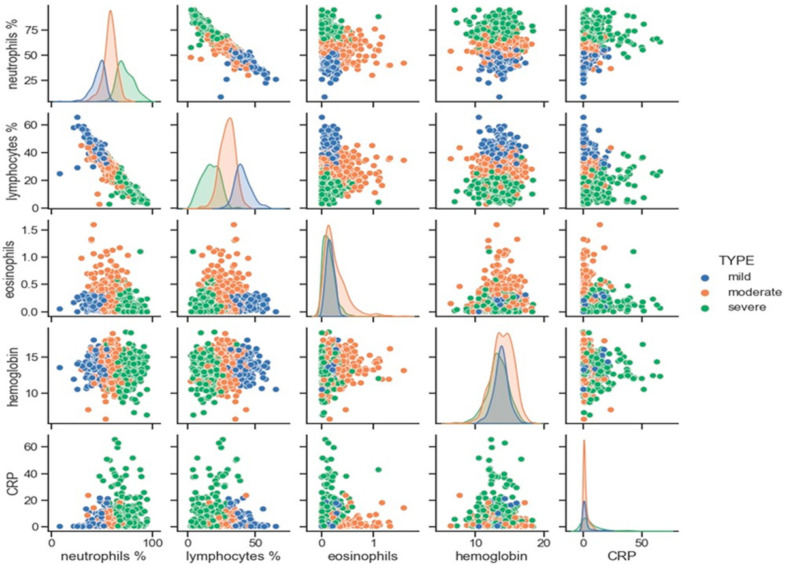
Diagonal plots represent the distribution of the target variable across the three patient clusters. The distribution of the individual values in pairs of variables for the three clusters are represented in scattered plots. Three clusters of patients: blue, light brown, and green represent mild, moderate, and severe bronchiectasis patients, respectively.

The five-dimensional space containing the patient data was reduced to a two-dimensional representation (Figure 2). Uniform Manifold Approximation and Projection (UMAP) was applied to transform the data into two linear combinations (UMAP1 and UMAP2, respectively) of the five target markers (blood parameters: neutrophils, lymphocytes, eosinophils, CRP, and hemoglobin) in which all the patients were represented in three colors (mild, moderate, and severe represented in blue, light brown, and green, respectively) [34] (Figure 2). Moreover, the distribution of the five target markers across the three patient clusters was also analyzed in the study as shown in Figure 3. The diagonal plots represented the distribution of the target variable across the three patient clusters. In addition, the distribution of the individual values in pairs of variables for all three clusters (blue, light brown, and green colors) were represented in scattered plots (Figure 3).

### 2.5. Statistical Analysis

The study variables are presented as mean (standard deviation) in tables. A subanalysis in which patients with COPD were excluded in all three clusters was also conducted. Potential differences among the three clusters of patients (cluster # 1, cluster # 2, cluster # 3), including those in which COPD patients were excluded, were assessed using one-way analysis of variance (ANOVA) and Tukey’s post hoc for the quantitative variables and the Chi-square test for the categorical variables. Correlations between clinical and biological variables were explored using the Pearson’s correlation coefficient. A Bonferroni-type adjustment was performed to considering the effect of having multiple correlations.

Correlations are displayed in graphical correlation matrixes, obtained from R package corrplot (https://cran.r-project.org/web/packages/corrplot/index.html, accessed on 2 December 2021), in different colors: blue for positive correlations and red for negative ones (Penn State University, World Campus, Pennsylvania, PA, USA).

Comparisons among the three patient clusters were also made on the basis of the degree of the disease severity according to the different scores (FACED, EFACED and BSI), in which the percentages of patients in each category were depicted. Potential differences among the three clusters were explored using the Chi-square test.

Multivariate ordinal logistic regression, in which the outcome variable was clusters (cluster # 1, cluster # 2, cluster # 3) was used to assess the potential associations of EFACED score with ordered clusters. The following clinically meaningful confounders were considered: Charlson index, COPD, platelets, ESR, fibrinogen, creatinine, total protein concentration, and albumin levels. The multivariate regression odds ratio (OR) is represented as a black dot in each of the confounders along with the corresponding confidence intervals, which were depicted in a forest plot. In the Y-axis, all the confounder variables are plotted, while in the X-axis, the width of the confidence intervals is represented. The one value is represented as a dotted vertical line. Statistical analyses were performed using SPSS 23.0 (SPSS Inc., Chicago, IL, USA) and Stata 15.1 (StataCorp LLC, College Station, TX, USA). Results were considered as statistically significant at a *p*-value < 0.05.

## 3. Results

### 3.1. General Clinical Characteristics of the Three Patient Clusters

**Figure 4:**
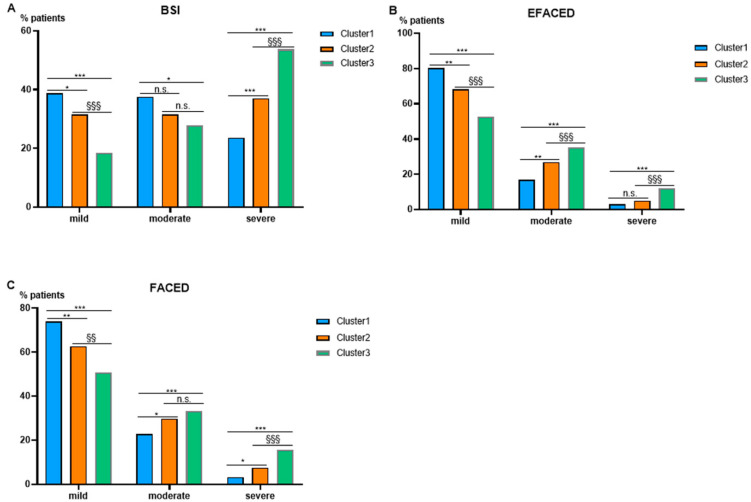
Histograms of the proportions of patients who were classified as mild, moderate, or severe according to BSI (**A**), EFACED (**B**) and FACED (**C**) among the three clusters. Score subdivisions were as follows: (1) BSI: mild: 0–4, moderate: 5–8, severe: ≥9, (2) EFACED: mild: 0–3, moderate: 4–6, severe: 7–9; and (3) FACED: mild: 0–2, moderate: 3–4, severe: 5–7. Statistical significance: *, *p ≤* 0.05; **, *p ≤* 0.01; ***, *p ≤* 0.001: Comparisons were assessed between either group # 3 or group # 2 and group # 1 (less severe); §§, *p ≤* 0.01; §§§, *p ≤* 0.001: Comparisons between groups # 3 and # 2. n.s.: no significance.

The number of female patients was greater than that of male patients in all three clusters (Table 2. Patients in cluster # 3 were significantly older than those represented in clusters # 1 and 2, respectively (Table 2). Disease severity scores FACED, EFACED, and BSI were significantly greater in cluster # 3 than in clusters # 1 and # 2 (Table 2). The proportions of cluster # 3 patients with mild and moderate degree of disease severity (BSI score) was significantly lower than in clusters # 1 and # 2 (mild, Figure 4A). Conversely, BSI score was greater in patients of clusters # 2 and # 3 than those in cluster # 1. Moreover, BSI score was also significantly higher in cluster # 3 than in cluster # 2 (Figure 4A). The proportions of patients with moderate and severe EFACED and FACED scores were significantly higher in cluster # 3 than in clusters # 1 and # 2 (EFACED), as illustrated in Figure 4B,C, respectively.

The number of exacerbations was significantly higher in cluster # 3 patients than in cluster # 1 (Table 2). The number of hospitalizations and Charlson index were significantly higher in cluster # 3 than in clusters # 1 and # 2 (Table 2). The proportions of patients with colonization by *Pseudomonas aeruginosa* and with concomitant COPD were greater in cluster # 3 patients than in cluster # 1 (Table 2). Smoking history did not significantly differ among the three clusters (Table 2). The degree of airflow limitation and diffusion lung capacity was significantly impaired in cluster # 3 patients compared to clusters # 1 and # 2 (Table 2). When COPD patients were excluded from the analysis, similar results were obtained in the three analyzed clusters (Table 3).

### 3.2. Systemic Inflammatory and Nutritional Parameters in the Three Cluster Patients

**Figure 5:**
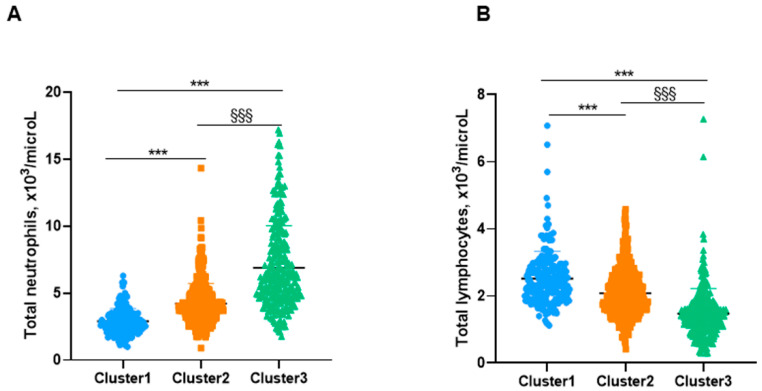
(**A**) Mean values and standard deviation of total number of neutrophils (10^3^/microL) among cluster 1, cluster 2, and cluster 3 in all the study patients. (**B**) Mean values and standard deviation of total number of lymphocytes (10^3^/microL) among cluster 1, cluster 2, and cluster 3 in all the study patients. Statistical significance: ***, *p* ≤ 0.001: Comparisons were assessed between either group # 3 or group # 2 and group # 1 (less severe §§§, *p* ≤ 0.001: Comparisons between groups # 3 and # 2.

**Figure 6:**
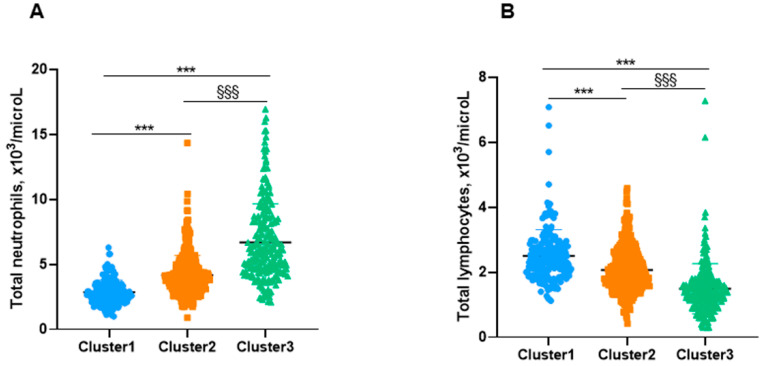
(**A**) Mean values and standard deviation of total number of neutrophils (10^3^/microL) in the three clusters of patients excluding those with COPD. (**B**) Mean values and standard deviation of total number of lymphocytes (10^3^/microL) in the three clusters of patients excluding those with COPD. Statistical significance: ***, *p* ≤ 0.001: Comparisons were assessed between either group # 3 or group # 2 and group # 1 (less severe §§§, *p* ≤ 0.001: Comparisons between groups # 3 and # 2.

**Figure 7:**
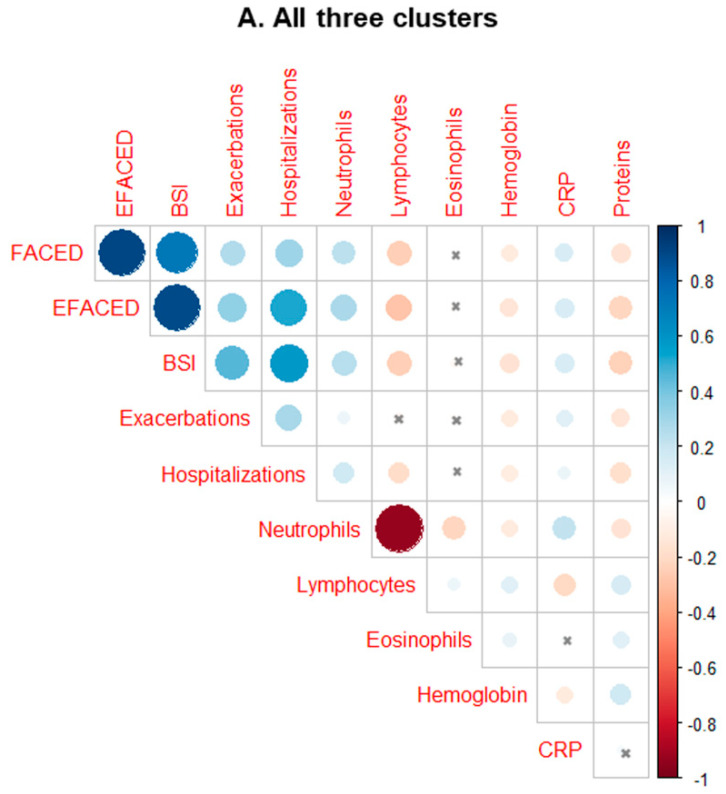

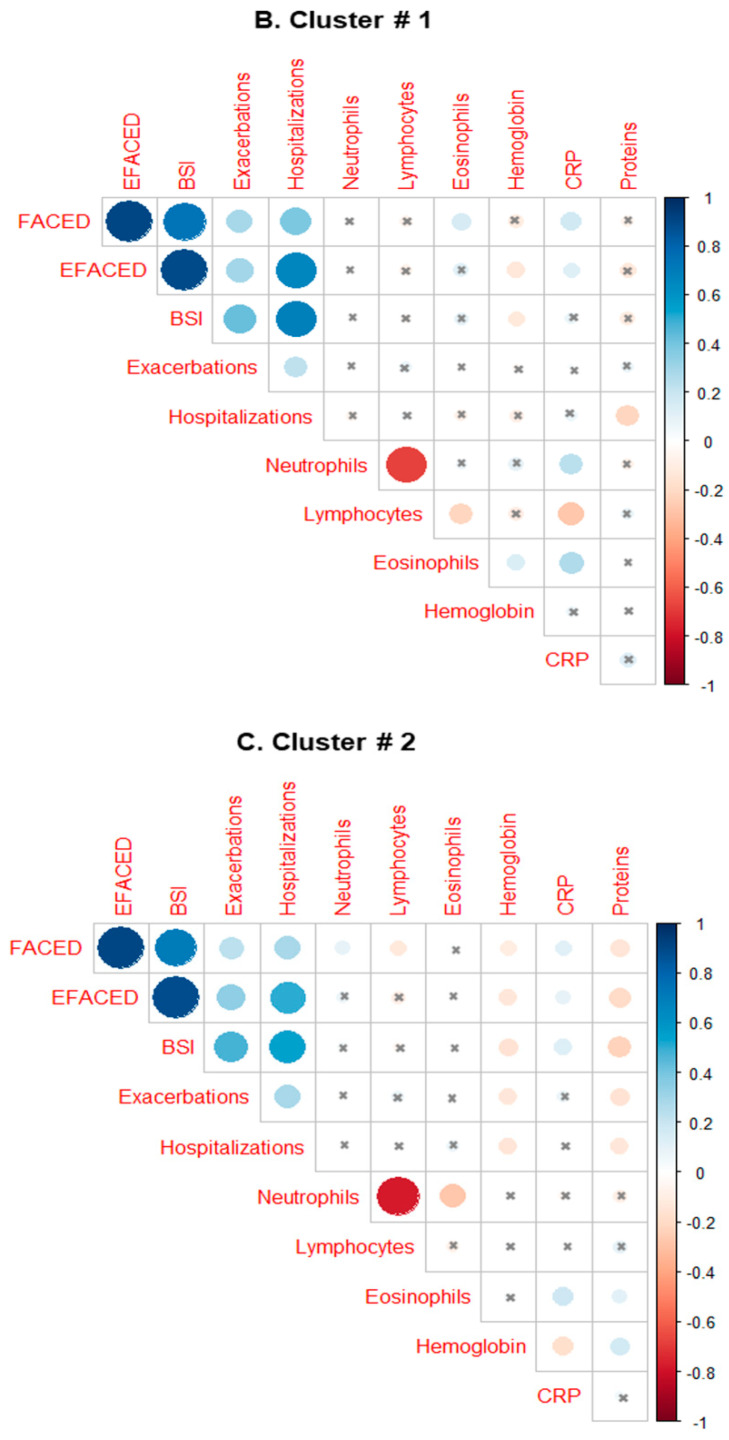

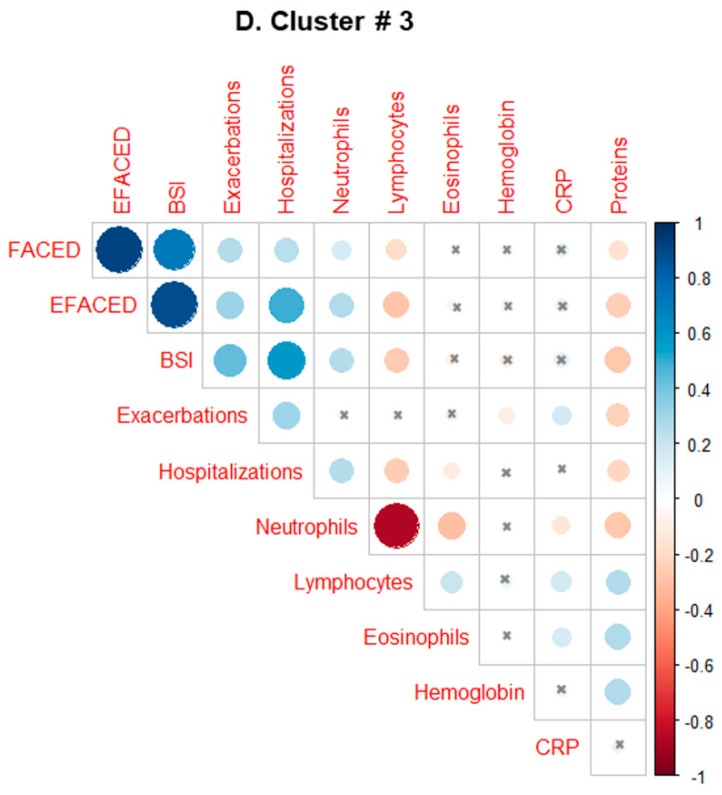
Correlation matrix of the disease severity and analytical variables, in which the positive correlations are represented in blue, while the negative correlations are represented in red: (**A**) all the study patients, (**B**) patients in cluster # 1, (**C**) patients in cluster # 2, and (**D**) patients in cluster # 3. The intersection within the circle represents a *p*-value > 0.05. The color intensity and the size of the circle are proportional to the correlation coefficients, as indicated in the Y-axis on the right-hand side of the graph.

Importantly, the levels of leukocytes and other inflammatory cells were significantly greater in cluster # 3 patients than in those represented in clusters # 1 and # 2 (Table 4 and Figure 5A,B and Figure 6A,B). Moreover, levels of other inflammatory parameters such as CRP, ESR, and fibrinogen were also significantly higher in cluster # 3 patients than in clusters # 1 and 2 (Table 4). CRP concentrations significantly and positively correlated with FACED, EFACED, BSI, exacerbations, hospitalizations, and neutrophil levels in all the patients as a whole (all three clusters, Figure 7A). Moreover, when patients from clusters # 1 and # 2, but not cluster # 3, were analyzed separately, significant associations were also detected between CRP levels and the variables FACED, EFACED, neutrophils, and eosinophils (cluster # 1) and FACED, EFACED, BSI, and eosinophils (cluster # 2), as depicted in Figure 7B,C, respectively. Protein levels inversely correlated with FACED, EFACED, BSI, exacerbations, hospitalizations, and neutrophil levels when patients were analyzed as a whole (clusters # 1–3) and when cluster # 3 was analyzed independently (Figure 7A,D, respectively). When cluster # 2 patients were analyzed independently, significant weaker correlations were observed between total protein levels and FACED, EFACED, BSI, and the number of exacerbations and hospitalizations (Figure 7C). The number of platelets along with hemoglobin and hematocrit levels was also greater in cluster # 3 patients than in clusters # 1 and # 2 (Table 4). Blood glucose levels were also higher in cluster # 3 patients than in clusters # 1 and # 2 (Table 4). Concentrations of total proteins and albumin, however, were significantly lower in cluster # 3 patients than in clusters # 1 and # 2 (Table 4). The analysis of the potential differences among the three clusters when COPD patients were excluded yielded similar results as shown in Table 5 and Figure 5A,B and Figure 6A,B.

### 3.3. Multivariate Analysis

**Figure 8:**
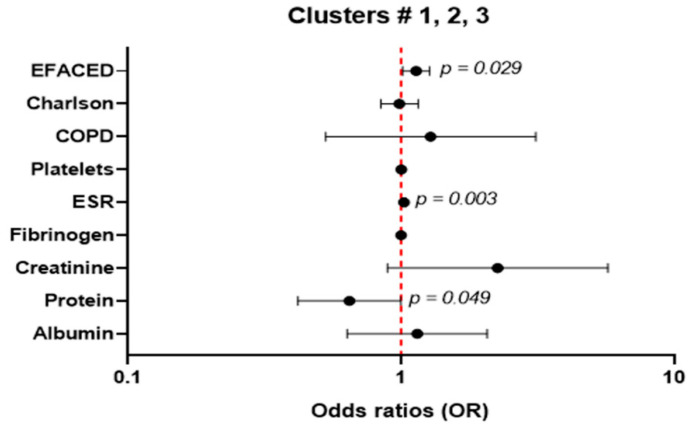
Multivariate ordinal logistic regression, in which the outcome variable was clusters (ordered from 1, the lowest level, to 3, the highest level) was used to assess the potential associations of EFACED score with each of the clusters. The following clinically meaningful confounders were considered: Charlson index, COPD, platelets, ESR, fibrinogen, creatinine, total protein concentration, and albumin levels. The multivariate regression odds ratio (OR) is represented as a black dot in each of the confounders along with the corresponding confidence intervals, which were depicted in a forest plot. In the Y-axis, all the confounder variables are plotted, while in the X-axis, the width of the confidence intervals is represented. The one value is represented as a dotted vertical line.

After adjusting for potential confounders, an association of EFACED score with clustered risk variable was demonstrated (OR = 1.136, CI 95%: 1.013–1.274; *p*-value = 0.029, Figure 6), demonstrating that patients with greater EFACED scores were more likely to fall within higher clusters (1–3). Moreover, associations of ESR and total protein levels with clustered risk variable were also demonstrated (OR = 1.025, CI 95%: 1.009–1.042; *p*-value = 0.003; OR = 0.647, CI 95%: 0.419–0.999; *p*-value = 0.049, respectively, Figure 8).

## 4. Discussion

Chronic respiratory diseases are characterized by a complex interaction between the airways pathobiology and systemic manifestations, such as inflammation and nutritional and metabolic abnormalities. Specifically, in a great number of bronchiectasis patients, microbial colonization plays a relevant role in the perpetuation of inflammatory events in the airways and systemic compartment of the patients, further contributing to increasing the number of exacerbations, disease progression, and prognosis [5,35,36]. Identification of the bronchiectasis patients who are placed at a greater risk to progress more rapidly and/or to experience exacerbations is still a major challenge in clinical settings. Currently available tools do not always help identify these specific groups of patients. Thus, other instruments need to be developed with the goal to define patterns of behavior from large data sets, which can be translated into well-structured groups of patients that can be specifically targeted in clinical settings. In the present study, using a data mining approach, five different clusters of patients have been identified in an initial stage. A refined analysis allowed us to merge a few of the clusters into three clinically different ones, whose specific clinical features have been analyzed from different standpoints. We believe that these are novel results that will have clinical implications.

Patients represented in cluster # 3 (*n* = 335) were older and had a significantly more severe disease as measured by all three disease scores (BSI, FACED, and EFACED) than patients in clusters # 1 and # 2. It is worth mentioning that in the three clusters, the behavior of patients with mild bronchiectasis was similar for all three indices (BSI, FACED, and EFACED). Nonetheless, in the group of patients with a moderate disease, the cluster distribution profile varied between the BSI score on the one hand and FACED and EFACED scores, on the other. The variables contributing to each specific score may account for the differences in disease severity distribution across the three identified clusters [30,31,32]. In addition, other important parameters such as Charlson index, the number of exacerbations and hospitalizations, the prevalence of COPD associated with bronchiectasis, and colonization by PA were also greater in cluster # 3 patients than in the other two clusters. These findings are in line with those recently reported in another investigation in which bronchiectasis patients were also subdivided into three major clusters on the basis of distinct airway phenotypic features [37,38]. The novelty in our investigation relies on the definition of three clinically relevant clusters based on the analysis of systemic variables such as neutrophils, lymphocytes, eosinophils, CRP, and hemoglobin.

Lung function parameters were also significantly impaired in cluster # 3 patients compared to patients classified in the other two clusters. Such an impairment in the degree of the airway obstruction and diffusion capacity was independent of the presence of COPD, as statistically significant differences were maintained among the three study clusters after excluding the COPD patients from the analysis in all three clusters. These results confirm that the differential phenotypes are associated with bronchiectasis per se rather than with COPD or the degree of the airflow limitation as also previously demonstrated [14,19].

A significant rise in the levels of cellular and molecular inflammatory parameters was observed in cluster # 3 patients compared to patients in clusters # 1 and # 2. These findings reveal that disease severity was probably associated with the degree of systemic inflammation in patients belonging to cluster # 3. In fact, significant positive associations were found between several inflammatory parameters (e.g., neutrophils and CRP) and disease severity scores as well as with the number of exacerbations and hospitalizations. Associations between disease severity and systemic inflammation have been previously reported [39,40,41]. The strong association between inflammation and disease progression and severity has also been confirmed in the present study.

Importantly, the differential phenotypic characteristics of the three groups of patients identified using a clustering approach were further confirmed by the multivariate ordinal logistic regression analysis, in which the EFACED score was associated with the clustered risk variable (OR: 1.136), indicating that patients in cluster # 3 were more severe than patients in the other two clusters. These are clearly novel findings that validate the methodological approaches used in the present study.

### Study Critique

A major strength in the investigation is the use of a large cohort of bronchiectasis patients from whom data have been obtained from 43 participating centers in Spain. Despite these advantages, several limitations also apply. The study of biological insights and disease pathogenesis would have required a different investigational approach, in which biological samples should be obtained from different compartments in the patients. The RIBRON, however, relies on clinical and analytical data obtained from the patients. Future investigations of different approaches based on the use of biological samples from the airways and lungs should be designed with the aim to elucidate specific mechanisms that are involved in the pathobiology of disease severity and progression in patients with bronchiectasis, especially in view of the profile of clinical and analytical parameters that have been analyzed in the current study. An external validation cohort aimed to further confirm the results reported herein would also be a matter of investigation in the near future. Differences between male and female patients were also analyzed previously in this registry [14]. Thus, these differences have not been analyzed in the present investigation. As abovementioned in the tables, the number of female patients was greater than that of male patients.

Another limitation is related to the lack of information on the potential colonization by pathogens other than PA in the airways and lungs of the study participants. As that information was not obtained from all the patients, it has not been analyzed in the present study.

Another limitation is related to the lack of additional analysis of biological parameters including cytokines (interleukin-6 and tumor necrosis factor-alpha) that could have been comprised in the cluster analysis. However, as those parameters were not collected in the RIBRON registry, they were not available for the cluster analysis. Future studies should aim to assess whether other systemic parameters may also contribute to differentiate several clinical phenotypes in patients with bronchiectasis.

## 5. Conclusions

Clustering analyses of systemic blood parameters identified three differential clinical phenotypes of bronchiectasis patients that were further confirmed by a logistic regression. These analyses revealed that disease severity was associated with the clusters, particularly with cluster # 3. Patients included in this group had a more severe disease, worse lung function, and a rise in systemic inflammatory parameters compared to patients in clusters # 1 and # 2. These findings were independent of the presence of COPD. Clustering analysis of systemic parameters offers a powerful tool to better characterize patients with bronchiectasis. These results have clinical implications in the management of the complexity and heterogeneity of bronchiectasis patients.

## Figures and Tables

**Table 1:** Characteristics of the five clusters resulting from the hierarchical clustering approach.

Cluster	Size	Average EFACED	Disease Severity	Summary of the Size	
1	367	2.60	moderate	**N = 242**	**Cluster # 1: mild**
2	311	3.57	severe	**N = 515**	**Cluster # 2: moderate**
3	24	3.67	severe	**N = 335**	**Cluster # 3: severe**
4	148	2.88	moderate		
5	242	2.13	mild		

As described in methods, the five clusters were transformed into three, which were the ones analyzed in the study. Definition of abbreviations: N, number.

**Table 2:** General characteristics of all the study patients according to cluster analyses.

	Cluster	Cluster	Cluster
	Group # 1	Group # 2	Group # 3
	N = 242	N = 515	N = 335
**Anthropometric variables**, $\bar{x}$(SD)			
Age, years	65.8 (14.9)	66.8 (15.1)	69.4 (14.7) *§
BMI, kg/m^2^	25.1 (4.6)	26.1 (5.0) *	25.8 (5.3)
Female, N/male, N	184/58	330/185	213/122
**Disease severity**, $\bar{x}$(SD)			
FACED score	1.64 (1.44)	2.05 (1.64) **	2.61 (1.82) ***§§§
EFACED score	2.13 (1.91)	2.68 (2.08) **	3.58 (2.33) ***§§§
BSI score	6.48 (4.32)	7.54 (4.54) *	9.31 (4.94) ***§§§
Exacerbations (mild/moderate)	1.33 (1.62)	1.57 (1.7)	1.72 (1.64) *
Hospitalization for exacerbations	0.37 (0.79)	0.65 (1.53) *	0.99 (1.44) ***§§
Charlson Index	1.58 (1.27)	1.89 (1.63) *	2.12 (1.69) ***§
Chronic colonization by PA, N (%)	46 (19)	135 (26.2) *	109 (32.5) ***
Radiological extension	2.8 (1.4)	2.9 (1.4)	2.9 (1.5)
COPD	18 (7.4%)	54 (10.5%)	49 (14.6%) **
Asthma	29 (12%)	45 (8.7%)	31 (9.3%)
**Smoking history**			
Never smokers, N (%)	149 (62)	313 (61)	193 (57.6)
Current smokers, N (%)	19 (8)	46 (9)	26 (8)
Ex-smokers, N (%)	74 (31)	156 (30)	116 (35)
Packs-year, $\bar{x}$(SD)	10.4 (20.7)	12.2 (22.3)	15.1 (25.5) §
**Lung function**, $\bar{x}$(SD)			
FEV_1_, % predicted	81 (24)	75 (24) **	67 (26) ***§§§
FVC, % predicted	88 (20)	86 (21)	81 (24) ***§§
FEV_1_/FVC, %	72 (12)	68 (12) **	65 (14) ***§§§
DL_CO_, % predicted	85 (17)	88 (25)	69 (21) ***§§§
K_CO_, % predicted	79 (31)	81 (40)	68 (37) §
RV, % predicted	139 (43)	135 (49)	144 (54)
TLC, % predicted	105 (18)	101 (19)	99 (22)
RV/TLC, %	50 (12)	49 (11)	53 (13) §

Continuous variables are presented as mean (standard deviation), while categorical variables are presented as the number of patients in each group along with the percentage for the study group. Definition of abbreviations: $\bar{x}$, mean; SD, standard deviation; N, number; kg, kilograms; m, meters; BMI, body mass index; FACED: F, FEV_1_; A, Age; C, Chronic colonization by *Pseudomonas aeruginosa*; E, radiologic extension; D, dyspnea; EFACED: FACED adding the exacerbation in the previous year; BSI: bronchiectasis severity index; FEV_1_, forced expiratory volume in the first second; FVC, forced vital capacity; RV, residual volume; TLC, total lung capacity; DLco, carbon monoxide transfer; K_CO_, Krogh transfer factor. Statistical analyses and significance: *, *p* ≤ 0.05; **, *p* ≤ 0.01; ***, *p* ≤ 0.001: Comparisons were assessed between either group # 3 or group # 2 and group # 1 (less severe); §, *p* ≤ 0.05; §§, *p* ≤ 0.01; §§§, *p* ≤ 0.001: Comparisons between groups # 3 and # 2.

**Table 3:** General characteristics in the two clusters of bronchiectasis patients excluding those with COPD.

	Cluster	Cluster	Cluster
	Group # 1	Group # 2	Group # 3
	N = 224	N = 461	N = 286
**Anthropometric variables**, $\bar{x}$(SD)			
Age, years	64.9 (14.9)	65.7 (15.3)	68.1 (15) *
BMI, kg/m^2^	25.2 (4.7)	25.9 (5.0)	25.8 (5.4)
Female, N/male, N	178/46	313/148	203/83
**Disease severity**			
FACED score	1.54 (1.38)	1.94 (1.58) **	2.38 (1.70) ***§§
EFACED score	2 (1.81)	2.54 (1.99) **	3.24 (2.18) ***§§§
BSI score	6.17 (4.05)	7.25 (4.45) **	8.58 (4.65) ***§§§
Exacerbations (mild/moderate)	1.3 (1.63)	1.57 (1.72)	1.61 (1.62)
Hospitalization for exacerbations	0.32 (0.68)	0.6 (1.49) *	0.89 (1.47) ***§
Charlson Index	1.55 (1.26)	1.81 (1.56)	2.02 (1.69) **
Chronic colonization by PA, N (%)	42 (18.8)	127 (27.5) *	88 (30.8) ***§
Radiological extension	2.8 (1.4)	2.9 (1.4)	3 (1.5)
**Smoking history**			
Never smokers, N (%)	146 (65.2)	309 (67)	192 (67.1)
Current smokers, N (%)	16 (7.1)	36 (7.8)	16 (5.6)
Ex-smokers, N (%)	62 (27.7)	116 (25.2)	78 (27.3)
Packs-year, $\bar{x}$(SD)	7.85 (16.27)	8.13 (17.61)	8.82 (17.34)
**Lung function**, $\bar{x}$(SD)			
FEV_1_, % predicted	86 (20)	78 (21) **	73 (23) ***§§
FVC, % predicted	92 (38)	88 (23)	85 (31) ***§
FEV_1_/FVC, %	74 (30)	71 (23) **	69 (27) ***§
DL_CO_, % predicted	90 (40)	93 (30)	77 (20) **§§§
K_CO_, % predicted	87 (1)	87 (1)	80 (1)
RV, % predicted	143 (74)	141 (41)	153 (36)
TLC, % predicted	108 (58)	103 (48)	101 (48) *
RV/TLC, %	53 (31)	50 (23)	57 (29) §

Continuous variables are presented as mean (standard deviation), while categorical variables are presented as the number of patients in each group along with the percentage for the study group. Definition of abbreviations: $\bar{x}$, mean; SD, standard deviation; N, number; kg, kilograms; m, meters; BMI, body mass index; FACED: F, FEV_1_; A, Age; C, Chronic colonization by *Pseudomonas aeruginosa*; E, radiologic extension; D, dyspnea; EFACED: FACED adding the exacerbation in the previous year; BSI: bronchiectasis severity index; FEV_1_, forced expiratory volume in the first second; FVC, forced vital capacity; RV, residual volume; TLC, total lung capacity; DLco, carbon monoxide transfer; K_CO_, Krogh transfer factor. Statistical analyses and significance: *, *p* ≤ 0.05; **, *p* ≤ 0.01; ***, *p* ≤ 0.001: Comparisons were assessed between either group # 3 or group # 2 and group # 1 (less severe); §, *p* ≤ 0.05; §§, *p* ≤ 0.01; §§§, *p* ≤ 0.001: Comparisons between groups # 3 and # 2.

**Table 4:** Systemic inflammatory and nutritional parameters in the study patients according to clusters analyses.

	Cluster	Cluster	Cluster
	Group # 1	Group # 2	Group # 3
	N = 242	N = 515	N = 335
Female, N/male, N	184/58	330/185	213/122
**Blood parameters**, $\bar{x}$(SD)			
Total leukocytes, ×10^3^/μL	6.2 (1.8)	7.2 (2.1) ***	9.2 (3.5) ***§§§
Total neutrophils, ×10^3^/μL	2.9 (0.9)	4.2 (1.5) ***	6.9 (3.1) ***§§§
Neutrophils, %	46.6 (6.7)	58 (6.4) ***	73.3 (8.3) ***§§§
Total lymphocytes, ×10^3^/μL	2.5 (0.8)	2.1 (0.7) ***	1.5 (0.8) ***§§§
Lymphocytes, %	41 (6.2)	29.4 (5.8) ***	16.9 (6.7) ***§§§
Total eosinophils, ×10^3^/μL	0.2 (0.1)	0.3 (0.2) ***	0.1 (0.1) §§§
Eosinophils, %	2.6 (1.3)	3.5 (2.9) ***	1.6 (1.5) ***§§§
Platelets, ×10^3^/μL	245.8 (68)	250.7 (70.5)	264.1 (86.7) *§
CRP, mg/dL	2 (4.2)	1.8 (2.8)	7.3 (11.4) ***§§§
ESR, mm/h	15.9 (13.5)	15 (14.1)	25.9 (22.2) ***§§§
Fibrinogen, mg/dL	388.1 (109.7)	414 (125.3)	482 (153.8) ***§§§
Hemoglobin, g/dL	13.7 (1.1)	13.9 (1.6)	13.2 (1.6) **§§§
Hematocrit, %	41.6 (3.2)	42.3 (4.5)	40.4 (4.7) **§§§
Glucose, mg/dL	94.2 (16)	97.9 (27)	110.8 (46.9) ***§§§
Creatinine, mg/dL	0.8 (0.5)	0.8 (0.2)	0.9 (0.6) §
Total proteins, g/dL	7.13 (0.54)	7.01 (0.62)	6.96 (0.66) *
Albumin, g/dL	4.27 (0.36)	4.22 (0.43)	4.11 (0.49) **§

Continuous variables are presented as mean (standard deviation) for the study group. Definition of abbreviations: $\bar{x}$, mean; SD, standard deviation; CRP, C-reactive protein; ESR, erythrocyte sedimentation rate; μL, microliter; dL, deciliter; mg, milligrams; mm, millimeters; h, hour; ng, nanogram. Statistical analyses and significance: *, *p* ≤ 0.05; **, *p* ≤ 0.01; ***, *p* ≤ 0.001: Comparisons were assessed between either group # 3 or group # 2 and group # 1 (less severe); §, *p* ≤ 0.05; §§§, *p* ≤ 0.001: Comparisons between groups # 3 and # 2.

**Table 5:** Systemic inflammatory and nutritional parameters in the three clusters of patients excluding those with COPD.

	Cluster	Cluster	Cluster
	Group # 1	Group # 2	Group # 3
	N = 224	N = 461	N = 286
Female, N/male, N	178/46	313/148	203/83
**Blood parameters**, $\bar{x}$(SD)			
Total leukocytes, ×10^3^/μL	6.12 (1.73)	7.09 (2.16) ***	8.95 (3.34) ***§§§
Total neutrophils, ×10^3^/μL	2.86 (0.93)	4.17 (1.51) ***	6.68 (2.99) ***§§§
Neutrophils, %	46.5 (6.7)	57.84 (6.45) ***	72.9 (8.1) ***§§§
Total lymphocytes, ×10^3^/μL	2.5 (0.81)	2.07 (0.66) ***	1.49 (0.77 )***§§§
Lymphocytes, %	41.14 (6.17)	29.5 (5.84) ***	17.34 (6.64) ***§§§
Total eosinophils, ×10^3^/μL	0.16 (0.08)	0.26 (0.23)	0.13 (0.12)
Eosinophils, %	2.62 (1.35)	3.56 (2.92) ***	1.65 (1.47) ***§§§
Platelets, ×10^3^/μL	246 (68)	252 (70)	267 (88) **§
CRP, mg/dL	1.89 (3.95)	1.78 (2.75)	7.09 (10.91) ***§§§
ESR, mm/h	15.82 (13.68)	15.31 (13.75)	25.8 (22.39) ***§§§
Fibrinogen, mg/dL	388 (112)	412 (127)	474 (154) ***§§§
Hemoglobin, g/dL	13.67 (1.04)	13.82 (1.55)	13.14 (1.54) ***§§§
Hematocrit, %	41.54 (3.1)	42.08 (4.32)	40.17 (4.48) **§§§
Glucose, mg/dL	95 (16)	97 (26)	108 (46) ***§§§
Creatinine, mg/dL	0.79 (0.56)	0.8 (0.22)	0.89 (0.61)§
Total proteins, g/dL	7.13 (0.54)	7.03 (0.61)	7.03 (0.6)
Albumin, g/dL	4.27 (0.37)	4.24 (0.42)	4.17 (0.44)

Continuous variables are presented as mean (standard deviation) for the study group. Definition of abbreviations: $\bar{x}$, mean; SD, standard deviation; CRP, C-reactive protein; ESR, erythrocyte sedimentation rate; μL, microliter; dL, deciliter; mg, milligrams; mm, millimeters; h, hour; ng, nanogram. Statistical analyses and significance: **, *p* ≤ 0.01; ***, *p* ≤ 0.001: Comparisons were assessed between either group # 3 or group # 2 and group # 1 (less severe); §, *p* ≤ 0.05; §§§, *p* ≤ 0.001: Comparisons between groups # 3 and # 2.

## Data Availability

The datasets generated and analyzed during the current study are available from the corresponding author on reasonable request.
